# Supplementary material for: Causal Relationship between Aging and Anorexia Nervosa: A White-Matter-Microstructure-Mediated Mendelian Randomization Analysis
Source: Biomedicines. 2024 Aug 16;12(8):1874. doi: 10.3390/biomedicines12081874 (PMC11351342; doi:10.3390/biomedicines12081874)
Supplement: Supplementary file 1 [file biomedicines-12-01874-s001.zip › Supplementary Figure/Figure S4/Singe SNP plot of MR analysis for TL to ubm-b-1559.pdf]

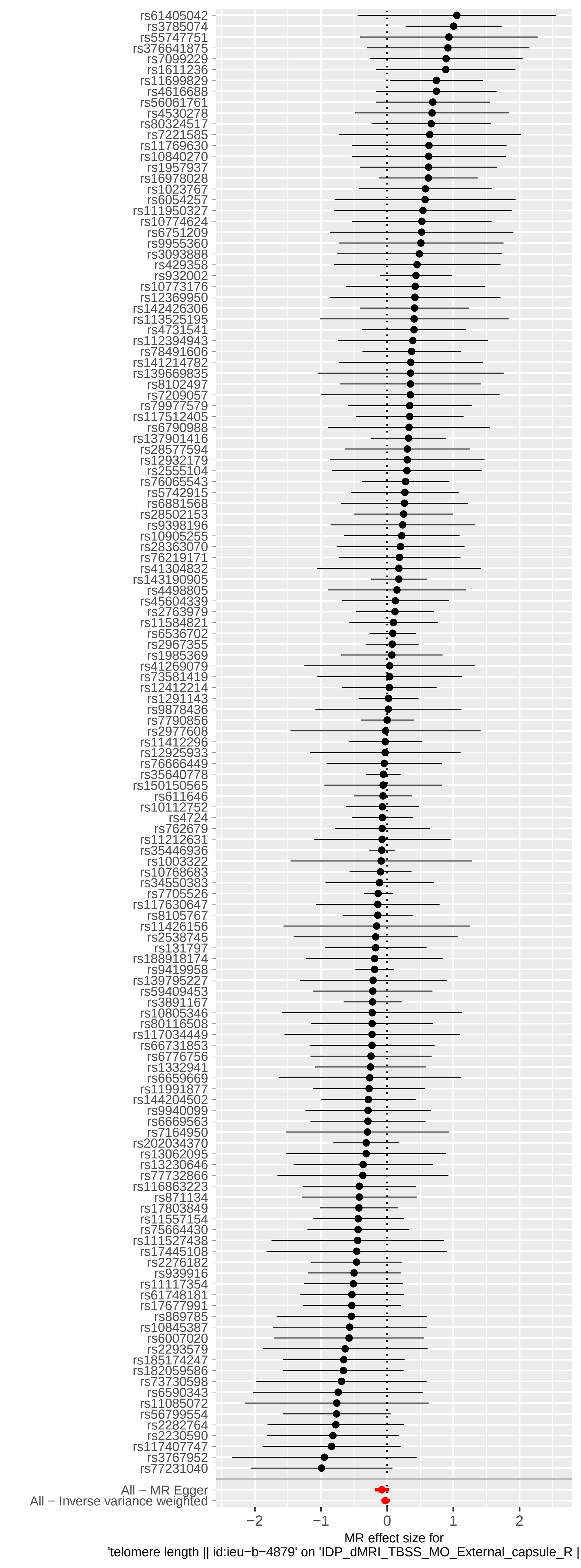
rs61405042
rs3785074
rs55747751
rs376641875
rs7099229
rs1611236
rs11699829
rs4616688
rs56061761
rs4530278
rs80324517
rs7221585
rs11769630
rs10840270
rs1957937
rs16978028
rs1023767
rs6054257
rs111950327
rs10774624
rs6751209
rs9955360
rs3093888
rs429358
rs932002
rs10773176
rs12369950
rs142426306
rs113525195
rs4731541
rs112394943
rs78491606
rs141214782
rs139669835
rs8102497
rs7209057
rs79977579
rs117512405
rs6790988
rs137901416
rs28577594
rs12932179
rs2555104
rs76065543
rs5742915
rs6881568
rs28502153
rs9398196
rs10905255
rs28363070
rs76219171
rs41304832
rs143190905
rs4498805
rs45604339
rs2763979
rs11584821
rs6536702
rs2967355
rs1985369
rs41269079
rs73581419
rs12412214
rs1291143
rs9878436
rs7790856
rs2977608
rs11412296
rs12925933
rs76666449
rs35640778
rs150150565
rs611646
rs10112752
rs4724
rs762679
rs11212631
rs35446936
rs1003322
rs10768683
rs34550383
rs7705526
rs117630647
rs8105767
rs11426156
rs2538745
rs131797
rs188918174
rs9419958
rs139795227
rs59409453
rs3891167
rs10805346
rs80116508
rs117034449
rs66731853
rs6776756
rs1332941
rs6659669
rs11991877
rs144204502
rs9940099
rs6669563
rs7164950
rs202034370
rs13062095
rs13230646
rs77732866
rs116863223
rs871134
rs17803849
rs11557154
rs75664430
rs111527438
rs17445108
rs2276182
rs939916
rs11117354
rs61748181
rs17677991
rs869785
rs10845387
rs6007020
rs2293579
rs185174247
rs182059586
rs73730598
rs6590343
rs11085072
rs56799554
rs2282764
rs2230590
rs117407747
rs3767952
rs77231040
All − MR Egger
All − Inverse variance weighted
−2
−1
0
1
2
MR effect size for
'telomere length || id:ieu−b−4879' on 'IDP_dMRI_TBSS_MO_External_capsule_R ||
